# A Novel Animal Model for Pulmonary Hypertension: Lung Endothelial-Specific Deletion of *Egln1* in Mice

**DOI:** 10.35534/jrbtm.2024.10007

**Published:** 2024-05-30

**Authors:** Bin Liu, Dan Yi, Xiaokuang Ma, Karina Ramirez, Hanqiu Zhao, Xiaomei Xia, Michael B. Fallon, Vladimir V. Kalinichenko, Shenfeng Qiu, Zhiyu Dai

**Affiliations:** 1 Division of Pulmonary, Critical Care and Sleep, University of Arizona, Phoenix, AZ 85004, USA; 2 Department of Internal Medicine, College of Medicine-Phoenix, University of Arizona, Phoenix, AZ 85004, USA; 3 Translational Cardiovascular Research Center, College of Medicine-Phoenix, University of Arizona, Phoenix, AZ 85004, USA; 4 Basic Medical Sciences, College of Medicine-Phoenix, University of Arizona, Phoenix, AZ 85004, USA; 5 Division of Neonatology, Phoenix Children’s Hospital, Phoenix, AZ 85016, USA; 6 Phoenix Children’s Health Research Institute, College of Medicine-Phoenix, University of Arizona, Phoenix, AZ 85004, USA; 7 BIO5 Institute, University of Arizona, Tucson, AZ 85721, USA; 8 Sarver Heart Center, University of Arizona, Tucson, AZ 85724, USA

**Keywords:** Pulmonary arterial hypertension, Hypoxia, TMEM100, Right heart, Endothelial cells

## Abstract

Pulmonary arterial hypertension (PAH) is a devastating disease characterized by high blood pressure in the pulmonary arteries, which can potentially lead to heart failure over time. Previously, our lab found that endothelia-specific knockout of *Egln1*, encoding prolyl 4-hydroxylase-2 (PHD2), induced spontaneous pulmonary hypertension (PH). Recently, we elucidated that *Tmem100* is a lung-specific endothelial gene using *Tmem100-CreERT2* mice. We hypothesize that lung endothelial-specific deletion of *Egln1* could lead to the development of PH without affecting *Egln1* gene expression in other organs. *Tm*em*100*-CreERT2 mice were crossed with *Egln1*^*flox/flox*^ mice to generate *Egln1*^*f/f*^*;Tmem100-CreERT2* (LiCKO) mice. Western blot and immunofluorescent staining were performed to verify the knockout efficacy of *Egln1* in multiple organs of LiCKO mice. PH phenotypes, including hemodynamics, right heart size and function, pulmonary vascular remodeling, were evaluated by right heart catheterization and echocardiography measurements. Tamoxifen treatment induced *Egln1* deletion in the lung endothelial cells (ECs) but not in other organs of adult LiCKO mice. LiCKO mice exhibited an increase in right ventricular systolic pressure (RVSP, ~35 mmHg) and right heart hypertrophy. Echocardiography measurements showed right heart hypertrophy, as well as cardiac and pulmonary arterial dysfunction. Pulmonary vascular remodeling, including increased pulmonary wall thickness and muscularization of distal pulmonary arterials, was enhanced in LiCKO mice compared to wild-type mice. *Tmem100* promoter-mediated lung endothelial knockout of *Egln1* in mice leads to development of spontaneous PH. LiCKO mice could serve as a novel mouse model for PH to study lung and other organ crosstalk.

## 1. Introduction

Pulmonary hypertension (PH) is a devastating lung disease characterized by high blood pressure in the pulmonary arteries, which has the potential to lead to heart failure over time. Endothelial cell (EC) dysfunction plays a significant role in the development of PH [1]. So far, many animal models have been established in mice to understand the role of key factors and genes in the ECs in the pathogenesis of PH. However, due to the ubiquitous expression of endothelial makers in all the organs, such as the pan-markers Cdh5 and Tie2, there are potential off-target effects in these pan-EC Cre mice models [2]. The goal of this study is to demonstrate a lung endothelial-specific Cre tool for lung endothelial-specific manipulation in PH.

The lung possesses a unique morphology characterized by a thin layer of capillary ECs that facilitate efficient gas exchange, in contrast to other organs. Despite their critical importance in lung function, a Cre line specific to lung ECs has not been identified in the literature. Recent analysis using single-cell RNA sequencing of murine ECs across various organs revealed Tmem100 as one of the most enriched genes specifically in lung vasculature compared to other organ vasculatures [3]. Our recent investigations employing immunostaining against TMEM100 in different human organs indicated high expression of TMEM100 exclusively in human lung ECs, with no detectable expression in ECs from other organs such as the heart, liver, and kidney. Our recent studies utilizing Tmem100-CreERT2; Ai6 mice illustrated predominant expression of Tmem100 (ZsGreen) in lung ECs of adult mice. Further analysis of Tmem100 (ZsGreen) expression in the lung demonstrated high expression in 92% of capillary ECs, 72% of arterial ECs, and 46% of venous ECs [4].

We previously developed a new mouse model displaying severe PH through *Tie2Cre*-mediated disruption of *Egln1*, which encodes hypoxia-inducible factor (HIF) prolyl hydroxylase 2 (PHD2). This model, designated as *Egln1*^*Tie2Cre*^, exhibits progressive obliterative vascular remodeling characterized by vascular occlusion and plexiform-like lesions, ultimately leading to right heart failure [5]. We aimed to establish a lung endothelial-specific deletion of *Egln1* PH model using *Tmem100-CreERT2*.

## 2. Methods and Materials

*Tmem100-CreERT2;Ai6* mice were generated previously [4]. *Egln1*^*f/f*^*;Tmem100-CreERT2* (LiCKO) mice were generated by crossing the *Egln1*^*flox/flox*^ (WT) mice (JAX # 009672) and *Tmem100*-*CreERT2* mice (JAX # 014159). *Egln1*^*f/f*^*;Cdh5-CreERT2* (iCKO) mice were generated by crossing the *Egln1*^*flox/flox*^ (WT) mice and *Cdh5-CreERT2* mice (from Ralf Adams) [6]. WT, LiCKO and iCKO mice aged 2–5 months were used in these studies. The animal care and study protocols were reviewed and approved by the Institutional Animal Care and Use Committees of the University of Arizona (#19–513).

### 2.1. Hemodynamic Measurement

Right ventricular systolic pressure (RVSP) was measured with a 1.4F pressure transducer catheter (Millar Instruments) and recorded with AcqKnowledge software (Biopac Systems Inc., Goleta, CA, USA) as described previously [7,8]. The catheter was inserted into the right ventricle in mice under anesthesia (100 mg ketamine/ 5mg xylazine /kg body weight, i.p.).

### 2.2. Echocardiography

Echocardiography was performed in the University of Arizona as described previously [9]. Transthoracic echocardiography was performed using a VisualSonics Vevo 3100 ultrasound machine (FujiFilm VisualSonics Inc., Toronto, ON, Canada) with an MS550D (40 MHz) transducer. The right ventricle wall thickness during diastole (RV WTD) was obtained from the parasternal short-axis view at the papillary muscle level using M-mode. The RV cross-sectional area was obtained from the parasternal short-axis view at the papillary muscle level using B-mode. Pulmonary artery (PA) acceleration time and PA ejection time were obtained from the parasternal short-axis view at the aortic valve level using pulsed Doppler mode. The cardiac output (CO) was obtained from the parasternal short-axis view using M-mode.

### 2.3. Whole Organ Imaging, Immunofluorescent Staining, and Histological Staining

Various organs from *Tmem100-CreERT2;Ai6* mice post-tamoxifen treatment at approximately 2 weeks were perfused by PBS, followed by dissection and imaging under the fluorescent stereomicroscope. Mouse lung tissues were perfused with PBS, inflated with 50% OCT in PBS, and embedded in 100% OCT for cryosectioning. For immunofluorescent staining on these fresh frozen tissues, lung sections (5 μm) were fixed with 4% paraformaldehyde and blocked with 0.1% Triton X-100 and 5% normal goat serum at room temperature for 1 h. After 3 washes with PBS, the slides were incubated with anti-CD31 antibody (BD Bioscience, San Jose, CA, USA, Cat#550274, 1:25), anti-PHD2 (Cell Signaling Technology, Danvers, MA, USA, Cat#3293, 1:50) at 4 °C overnight, then incubated with Alexa 594 or 647-conjugated anti-rat or anti-rabbit IgG (Thermo Fisher Scientific, Waltham, MA, USA) at room temperature for 1 h. Nuclei were counterstained with DAPI mounting medium (SouthernBiotech, Birmingham, AL, USA).

For distal PA muscularization assessment, tissue slides were incubated with anti-α-SMA (Abcam, Cambridge, UK, Cat #ab5694, 1:300) at 4 °C overnight followed by Alexa 594 conjugated anti-rabbit IgG at room temperature for 1 h. Nuclei were counterstained with DAPI. Images were taken using Zeiss LSM710 confocal microscopy. α-SMA^+^ vessels were quantified blindly in 40 fields per lung.

Mouse lung tissues were perfused with PBS and fixed with 10% formalin via tracheal instillation at a constant pressure (15 cm H_2_O) and embedded in paraffin wax. Lung sections were stained with a Russel-Movat pentachrome staining kit (Cat #KTRMP, StatLab) according to the manufacturer’s protocol. For assessment of PA wall thickness, PAs from 40 images at 20X magnification were quantified blindly by Image J. Wall thickness was calculated by the distance between internal wall and external wall divided by the distance between external wall and the center of lumen.

### 2.4. Western Blot

Mice from various organ tissues were collected in RIPA lysis buffer supplemented with protease inhibitor cocktails (Sigma-Aldrich, St. Louis, USA). Approximately 20 μg of protein was loaded onto SDS-PAGE gels for Western Blotting. PVDF membranes were probed with anti-PHD2 (Cell Signaling Technology, Danvers, MA, USA, Cat #3293, 1:1000) or anti-β-actin (Sigma-Aldrich, St. Louis, MO, USA, Cat #A2228, 1:10,000) or GADPH (Sigma-Aldrich, St. Louis, MO, USA, Cat #G8795, 1:10,000) antibodies.

### 2.5. Statistical Analysis

Statistical analysis was performed using Prism 9 (Graphpad Software Inc., La Jolla, CA, USA). Statistical significance was determined by one-way ANOVA with Tukey’s post hoc analysis, calculating *p*-values corrected for multiple comparisons. Two-group comparisons were analyzed using unpaired two-tailed *t*-tests. A *p*-value less than 0.05 indicated a statistically significant difference. All bar graphs represent mean ± SD.

## 3. Results

### 3.1. Tmem100-CreERT2 Mediates Lung Endothelial-Specific PHD2 Deletion

**Figure 1.**
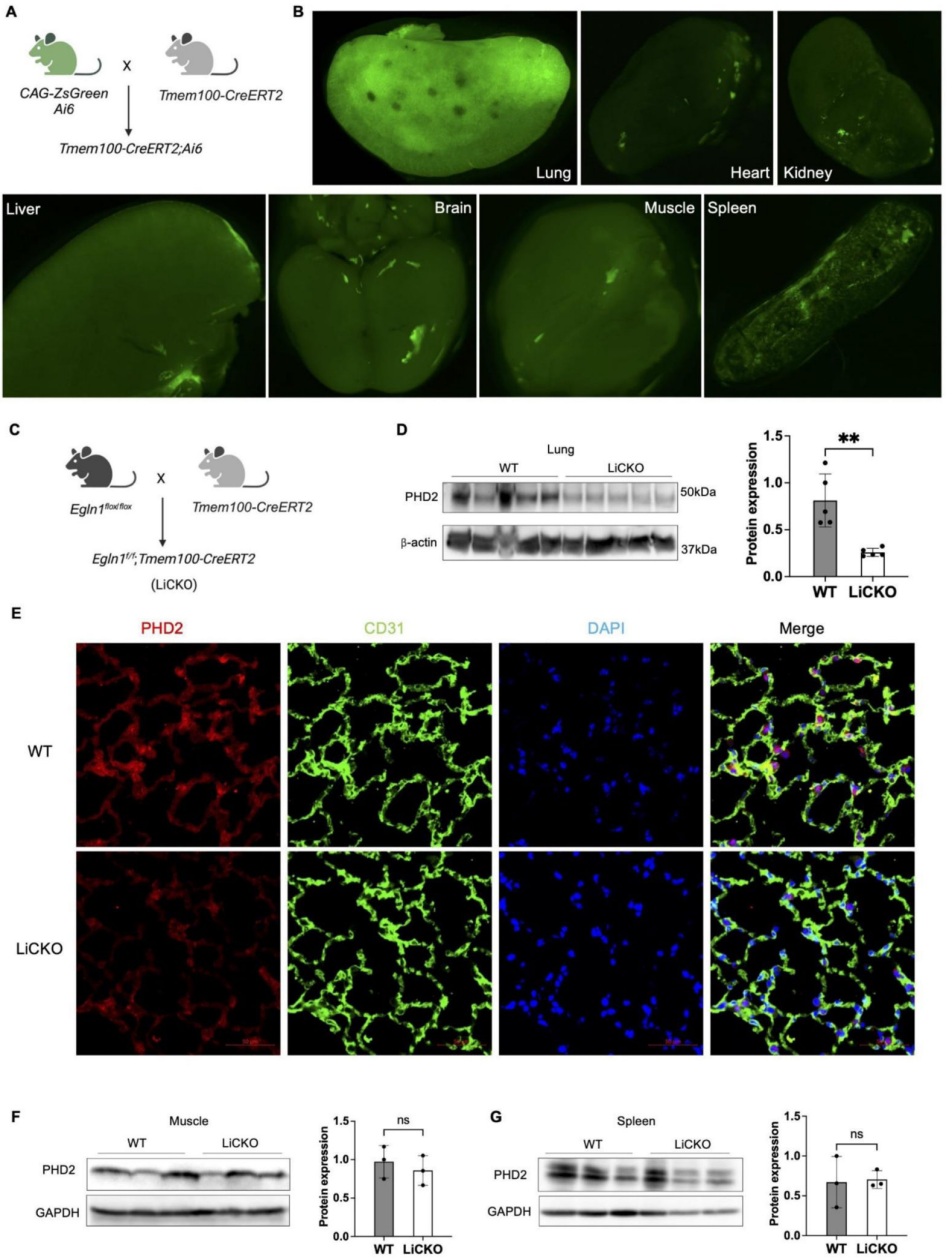
Lung endothelial-specific deletion of PHD2 in mice. (**A**) A diagram illustrating the strategy for generating lung endothelial-specific CreERT2 mice. (**B**) Whole organ imaging demonstrating the selective labeling of the lung by Tmem100-CreERT2 mice at the adult stage. ZsGreen indicates the presence of Tmem100 expression in organs. (**C**) A diagram illustrating the generation of lung endothelial-specific deletion of *Egln1* PH mouse model (*Egln1*^*f/f*^*;Tmem100CreERT2,* LiCKO). (**D**) Western blotting showed the knockdown of PHD2 expression in LiCKO lungs compared to WT (*Egln1*^*flox/flox*^) lungs. (**E**) Immunostaining showing the knockdown of PHD2 in lung capillary ECs compared to WT lungs. Green signals indicate CD31^+^ ECs. Red signals indicate PHD2 expression. (**F**) Western blotting showed that PHD2 expression was not affected in the skeletal muscle and spleen in LiCKO mice compared to WT mice. Student *t* test (**D**,**F**,**G**). **p < 0.01; ns, not significant. Scale bar: 50 μm (**E**).

Employing single-cell RNA sequencing analysis and mice lineage tracing approaches, our previous studies demonstrated that Tmem100 is a gene specifically expressed in lung ECs. *Tmem100-CreERT2* mediated ZsGreen expression (*Tmem100-CreERT2;Ai6*) in mice was mostly restricted to the lung vasculature compared to other organs, including the brain, heart, kidney and liver, as shown by immunofluorescent staining [4]. In this study, we applied whole organ imaging using fluorescent stereoscopy on tissues isolated from *Tmem100-CreERT2;Ai6* mice treated with tamoxifen (Figure 1A). We found that ZsGreen expression was highly present in the lung tissue and weakly observed in the heart, brain, kidney, spleen, muscle from isolated tissues (Figure 1B).

To further determine whether *Tmem100-CreERT2* can mediate genetic deletion mainly in lung ECs in vivo, we crossbred Tmem100-CreERT2 mice with *Egln1*^*flox/flox*^ mice to generate *Egln1*^*f/f*^;*Tmem100-CreERT2* (LiCKO) mice (Figure 1C). The LiCKO mice at the age of 7 weeks were injected with tamoxifen, and lung tissue was collected after one month. Western blotting analysis showed that lung PHD2 (encoded by *Egln1*) was markedly reduced in the LiCKO lungs compared to WT lungs (Figure 1D). Furthermore, immunostaining against PHD2 and the endothelial marker CD31 confirmed that PHD2 was reduced in the lung ECs of LiCKO compared to WT (Figure 1E). Moreover, we also collected major organs from the mice, including the heart, spleen, and skeleton muscle for western blotting analysis. Our data showed no expression change in PHD2 in the heart, spleen, and skeleton muscle (Figure 1F). Taken together, *Tmem100-CreERT2* mediated lung endothelial PHD2 deletion was restricted in the lung ECs.

### 3.2. Tmem100-CreERT2 Mediated Lung Endothelial PHD2 Deletion Induces Spontaneous PH in Mice

**Figure 2.**
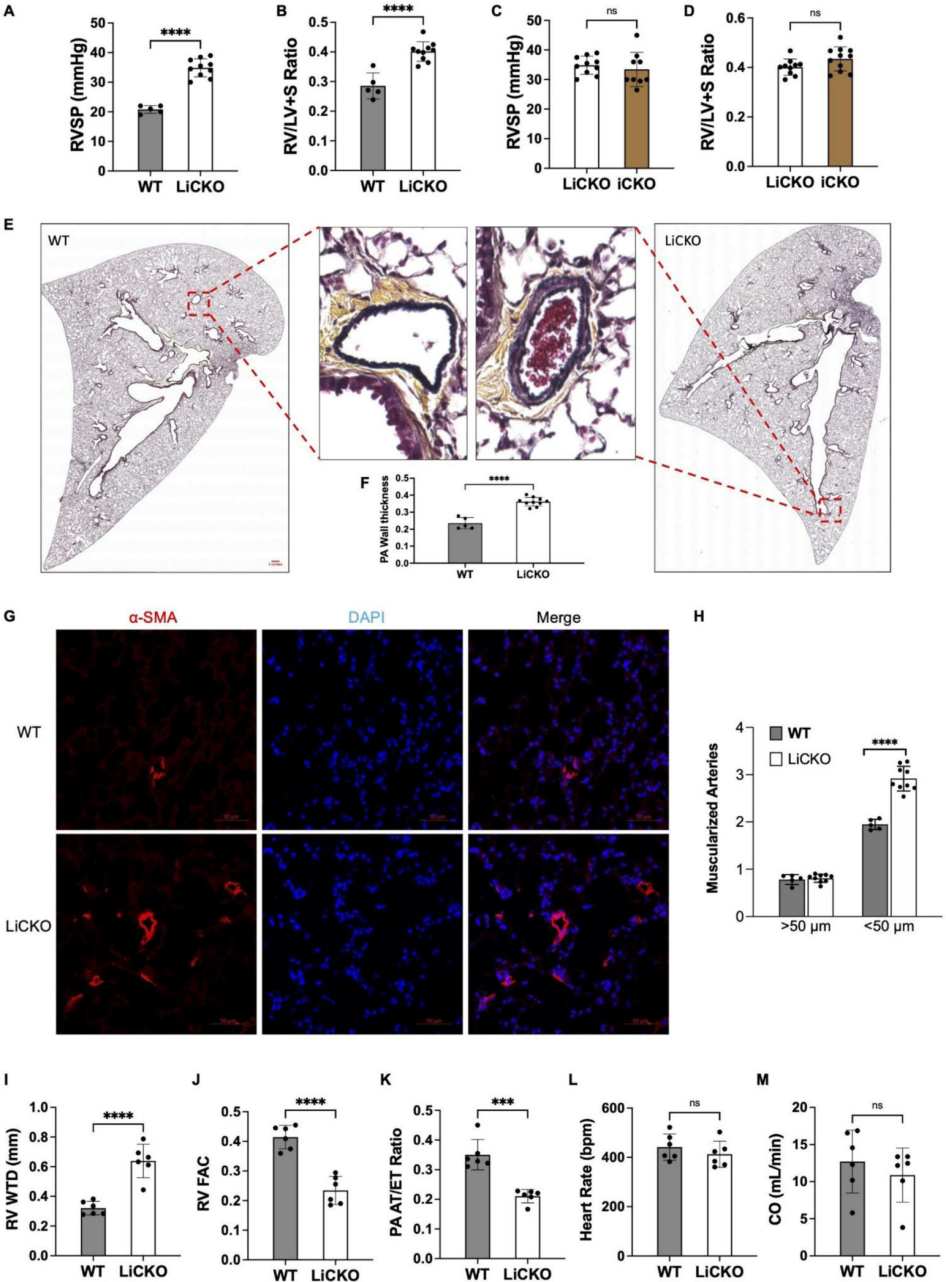
Lung endothelial-specific deletion of PHD2 induced PH and right heart dysfunction in mice. (**A**) Hemodynamic measurements showed that LiCKO mice developed elevated RVSP compared to WT mice after tamoxifen treatment. (**B**) Cardiac dissection revealed an increase in the RV/LV+S ratio, indicative of right heart hypertrophy, in LiCKO mice. (**C**,**D**) LiCKO mice developed similar degree of PH phenotypes compared to iCKO mice **(***Egln1*^*f/f*^*;Cdh5-CreERT2*). (**E**,**F**) Representative images from Russel-Movat Pentachrome staining and quantification of PA wall thickness demonstrated an increase in pulmonary vascular remodeling in LiCKO lungs compared to WT lungs. (**G**,**H**) Exemplary images and quantification data showed an increase in the muscularization of distal pulmonary vessels in LiCKO mice. Lung sections were subjected to immunostaining using anti-smooth muscle α-actin (SMA). (**I**) Echocardiography measurements revealed an increase in RV wall thickness during diastole (RV WTD) in LiCKO mice compared with WT mice. (**J**) Impaired RV fraction area change (RV FAC), indicating enhanced RV contractility, in LiCKO mice compared with WT mice. (**K**) An increased ratio of pulmonary artery acceleration time to ejection time (PA AT/ET) in LiCKO mice compared with WT mice. (**L**,**M**) There is no significant change of heart rate (HR), cardiac output (CO) between WT and LiCKO mice. Student *t* test (**A–D, I–M**). ***p < 0.001; ****p < 0.0001; ns, not significant. Scale bar: 50 μm (**G**).

Our previous studies and those of other groups have demonstrated that the loss of *Egln1* in ECs using *Tie2-Cre* and *Cdh5-Cre* or *Cdh5-CreERT2* mice induced spontaneous PH [5,10–12]. To determine whether LiCKO mice also develop PH, 7-week-old WT and LiCKO mice were challenged with tamoxifen for five consecutive days, followed by hemodynamic measurement at 1-month post-tamoxifen treatment. Hemodynamic data showed that the right ventricular systolic pressure (RVSP) was approximately 35 mmHg in LiCKO mice compared to 20 mmHg in WT mice (Figure 2A). The ratio of RV to left ventricular plus septum (RV/LV+S) was also significantly increased up to 0.45 in LiCKO mice compared to 0.25 in WT mice (Figure 2B). We then compared LiCKO mice with *Egln1*^*f/f*^*;Cdh5-CreERT2* (iCKO) mice after tamoxifen treatment and did not observe any difference in RVSP and RV/LV+S, suggesting that the efficacy of *Tmem100-CreERT2* is similar as *Cdh5-CreERT2* in inducing PH development. (Figure 2C,D)

We then characterized the pulmonary vascular remodeling in LiCKO mice by measuring pulmonary wall thickness based on the Pentachrome staining and distal pulmonary arterial muscularization based on α-smooth muscle actin (α-SMA) staining. Our data showed significant increases in pulmonary wall thickness and distal muscularized pulmonary vessels (<50 μm) in LiCKO mice compared to WT mice (Figure 2E,F). These data suggest that *Tmem100-CreERT2* mediated *Egln1* deletion induces spontaneous PH in mice.

Patients with PAH largely die due to right heart failure [13]. We next performed echocardiography measurements to determine whether LiCKO mice develop right heart dysfunction. Our echocardiography measurements showed that the LiCKO exhibited significant increases in RV wall thickness during diastole (RV WTD) (Figure 2G), while a decrease in PA function, assessed by PA acceleration time/ejection time (AT/ET) ratio (Figure 2H), and the right ventricular fractional area changes (RV FAC), indicative of RV contractility (Figure 2I). We did not observe any difference in cardiac output (CO) and heart rate (HR) between LiCKO mice and WT mice (Figure 2J,K). Taken together, these data suggest that *Tmem100-CreERT2* mediated *Egln1* deletion induces spontaneous PH and right heart dysfunction in mice.

## 4. Discussion

The present studies demonstrated that *Tmem100-CreERT2* mediated deletion of *Egln1* specifically causes the decrease of PHD2 in lung ECs but not in other organs. Loss of *Egln1* in lung ECs leads to spontaneous PH and right heart dysfunction.

*Tie2-Cre* and *Cdh5-Cre/CreERT2* are the wildly used pan-EC mouse models. However, there are potential off-target effects of these pan-EC mouse models, which restrict their use in lung vascular research. For example, pan-endothelial deletion of β-catenin in mice (*Ctnnb1*^*f/f*^*;EndoSCL-CreERT2*) led to blood-brain barrier leakage and lethality shortly after gene deletion induced by tamoxifen [14], preventing the utilization of this model in lung vasculature studies. Our study demonstrated that Tmem100-CreERT2 mediated PHD2 deletion is restricted to the lung but not to other organs. We also compared the efficacy of *Tmem100-CreERT2* with *Cdh5-CreERT2* mice and showed the similar effects in inducing PH development.

In the adult stage, Tmem100 exhibits its highest expression levels in the lung, with comparatively lower expression in the brain, heart, and muscle, as revealed by northern blot analysis [15]. Additional investigations have revealed its significant presence in the bone, esophagus, PaS cells, and proliferating zone chondrocytes through the utilization of reporter mice [16,17]. Our recent exploration, utilizing Tmem100-CreERT2; Ai6 mice, has unveiled predominant expression of Tmem100 (ZsGreen) in lung ECs, the right atrium, and cardiac valves in adult mice [4,18]. However, existing Tmem100-related mouse models have limitations as they were solely investigated in specific organs or developmental stages to assess Tmem100 expression. There remains a possibility that Tmem100 expression or depletion occurs in a spatiotemporal fashion within particular cells.

Accumulating evidence shows that PH is a systemic metabolic disease [19–21]. PH primarily leads to right-sided heart failure, triggering a multifaceted clinical syndrome that impacts various organ systems such as the left heart, brain, kidneys, liver, gastrointestinal tract, skeletal muscle, as well as the endocrine, immune, and autonomic systems. Understanding the interorgan crosstalk and interdependent mechanisms will help reduce the mortality and morbidity in patients.

Thus, *Tmem100-CreERT2* mouse model might be an ideal tool for dissecting the role of lung-specific ECs without affecting vascular beds in organs outside the lung. LiCKO mice primarily affect lung vasculature and PH development, which might be a novel model to investigate interorgan crosstalk in PH pathogenesis.
